# Identification of circulating microRNA signatures as potential biomarkers in the serum of elk infected with chronic wasting disease

**DOI:** 10.1038/s41598-019-56249-6

**Published:** 2019-12-23

**Authors:** Jessy A. Slota, Sarah J. Medina, Megan Klassen, Damian Gorski, Christine M. Mesa, Catherine Robertson, Gordon Mitchell, Michael B. Coulthart, Sandra Pritzkow, Claudio Soto, Stephanie A. Booth

**Affiliations:** 1 Zoonotic Diseases & Special Pathogens, Public Health Agency of Canada, National Microbiology Laboratory, 1015 Arlington St., Winnipeg, MB R3E 3R2 Canada; 2 Department of Medical Microbiology and Infectious Diseases, Faculty of Health Sciences, University of Manitoba, 730 William Ave., Winnipeg, MB R3E 0W3 Canada; 3 Canadian Creutzfeldt-Jakob Disease Surveillance System, Centre for Foodborne, Environmental and Zoonotic Infectious Diseases, Public Health Agency of Canada, Ottawa, ON K1A 0K9 Canada; 4 Mitchell Center for Alzheimer’s Disease and Related Brain Disorders, Department of Neurology, University of Texas Health Science Center at Houston, 6431 Fannin St., Houston, Texas 77030 USA; 5 National and OIE Reference Laboratory for Scrapie and CWD, Canadian Food Inspection Agency, Ottawa Laboratory Fallowfield, Ottawa, ON K2H 8P9 Canada

**Keywords:** Pathogens, Gene expression analysis

## Abstract

Chronic wasting disease (CWD) is an emerging infectious prion disorder that is spreading rapidly in wild populations of cervids in North America. The risk of zoonotic transmission of CWD is as yet unclear but a high priority must be to minimize further spread of the disease. No simple diagnostic tests are available to detect CWD quickly or in live animals; therefore, easily accessible biomarkers may be useful in identifying infected animals. MicroRNAs (miRNAs) are a class of small, non-coding RNA molecules that circulate in blood and are promising biomarkers for several infectious diseases. In this study we used next-generation sequencing to characterize the serum miRNA profiles of 35 naturally infected elk that tested positive for CWD in addition to 35 elk that tested negative for CWD. A total of 21 miRNAs that are highly conserved amongst mammals were altered in abundance in sera, irrespective of hemolysis in the samples. A number of these miRNAs have previously been associated with prion diseases. Receiver operating characteristic (ROC) curve analysis was performed to evaluate the discriminative potential of these miRNAs as biomarkers for the diagnosis of CWD. We also determined that a subgroup of 6 of these miRNAs were consistently altered in abundance in serum from hamsters experimentally infected with scrapie. This suggests that common miRNA candidate biomarkers could be selected for prion diseases in multiple species. Furthermore, Kyoto Encyclopedia of Genes and Genomes (KEGG) pathway analyses pointed to a strong correlation for 3 of these miRNAs, miR-148a-3p, miR-186-5p, miR-30e-3p, with prion disease.

## Introduction

Chronic Wasting Disease (CWD) is a prion disorder affecting captive and free-ranging cervids, including white-tailed deer, mule deer, elk, moose and reindeer^1^. Affected animals have been detected in 26 US states and 3 Canadian provinces, as well as Scandinavia and South Korea^2^. The disease is caused by abnormally folded prion proteins that induce conversion of the normal and noninfectious cellular form of the host prion protein (PrP^C^) into a misfolded infectious form, referred to as PrP^Sc ^^3,4^. The misfolding of PrP^C^ is seeded by PrP^Sc^, and this process enables prion replication and spread, particularly in tissues where PrP^C^ is highly expressed such as the central nervous system (CNS)^5^. The accumulation of PrP^Sc^ within the brain is associated with neuronal death, astrocytic gliosis and spongiform degeneration, ultimately causing a progressive decline in neurological function^6^.

Following a prolonged incubation period, animals infected with CWD often present with behavioral changes, altered movement and progressive weight loss, although these changes are often subtle early in the disease course^7^. Infectious PrP^Sc^ has been detected in body fluids such as blood, saliva, urine and feces, facilitating extensive shedding of prions into the environment^8^. Moreover, CWD prions have been shown to bind readily to soil particles and plant materials, allowing them to persist in the environment and contribute to animal-to-animal transmission^7,8^. In light of the emergence of this pervasive and highly transmissible disease, a current public health concern is to determine the risk for prion transmission to humans and livestock. Experimental studies and human prion disease surveillance suggest that, at least to date, the risk of CWD transmission to humans has probably been low. However, the degree of uncertainty remains high enough that precautionary steps are justified to control the spread of the disease in wild cervids, and limit human exposure to CWD^9^.

Diagnosis of CWD is typically performed postmortem by detection of PrP^Sc^ using either immunohistochemistry (IHC), enzyme-linked immunosorbent assay (ELISA) or western blotting^7^. The tissues used generally include the obex region of the brain and the retropharyngeal lymph nodes (RLN), and so samples taken in the wild must be shipped to approved laboratories for testing^10,11^. The relatively new methods of protein misfolding cyclic amplification (PMCA) and real-time quaking induced conversion (RT-QuIC) can both identify CWD prions with high sensitivity in various tissues from infected animals, as well as in some environmental samples^12–14^. CWD prions are widely distributed in peripheral tissues and biological fluids including blood, saliva, urine and feces^15–18^. Blood serum is often a peripheral fluid of choice in the development of diagnostic tests, owing to its accessibility. The detection of PrP^Sc^ using these accessible tissues and fluids may facilitate the development of rapid tests that could be implemented either in antemortem screening of farmed animals, or in the field to reduce hunter exposure to CWD. PMCA for example has been shown to provide accurate diagnosis of CWD in white-tailed deer that exhibit clinical signs of CWD (wasting), with 100% correlation with IHC positivity in brain and lymph nodes^19^. On the other hand, the diagnostic sensitivity of this test was 53% in early-stage symptomatic animals that were IHC-positive only in lymph nodes. Given that the asymptomatic stage of CWD lasts many months, novel serum biomarkers may be helpful in improving CWD diagnosis.

MicroRNAs (miRNAs) are short, non-coding RNAs that regulate gene expression through the RNA-interference pathway^20^. They can play crucial roles regulating cellular homeostasis, and their expression is known to be dysregulated by cellular stress and in various disorders and diseases^21^. Dysregulation of miRNAs has been shown in various prion diseases of both humans and animals in brain tissue as well as blood and CSF; however, to date no such studies have been reported for CWD. The majority of comprehensive studies have been performed using mouse models of scrapie and sCJD^22–29^. Other studies include BSE-infected macaques and patients with sporadic and genetic forms of Creutzfeldt-Jakob disease^22,23,30^. Despite differences in the platforms used for their analysis, a number of miRNAs were found to be altered in abundance in two or more studies, most notably miR-146a-5p, miR-16-5p, miR-124-3p, miR-26a-5p, miR-132-3p and miR-342-3p, suggesting these changes are indicative of common disease mechanisms. Interestingly, several miRNAs are known to regulate the expression of PrP^C^, potentially contributing to disease pathogenesis^31^. Due to their small size (18–25 nucleotides) and compact tertiary structures, miRNAs are resistant to nuclease digestion and are stable in circulating biofluids such as serum, saliva and cerebrospinal fluid. Importantly, miRNA abundance has been shown to be stable in whole blood, plasma and serum stored under a variety of conditions^32^. To date, no studies have been reported that investigate the global alterations in circulating miRNAs during prion diseases. However, an altered level of miR-342-3p was detected in the plasma of scrapie infected sheep by qRT-PCR, implying that disease-related miRNA signatures may also be found in blood^33^. Furthermore, circulating miRNAs have already been identified as biomarkers of other neurodegenerative diseases with pathologies and clinical presentations similar to prion infection, such as Alzheimer’s disease (AD) and Parkinson’s disease (PD)^34,35^.

In this study we used Illumina next-generation sequencing (NGS) to perform a global analysis of the miRNA content of serum from 35 elk that tested positive for CWD by IHC staining of PrP^Sc^ in obex tissue. Analysis revealed 47 miRNAs that were altered in these elk compared to 35 elk that were negative for CWD by IHC. Among these altered miRNAs, 21 showed potential as a diagnostic signature to discriminate CWD positive elk. Logistic regression models built using the normalized read count of these miRNAs were able to accurately predict CWD status in elk. Furthermore, in a small group of hamsters experimentally infected with hamster-adapted scrapie we found 6 of these miRNAs to be similarly affected. To our knowledge, this is the first demonstration of the use of high-throughput sequencing technologies to determine circulating miRNA alterations in any animal suffering from a prion disease. These miRNAs may serve as the basis of future non-invasive diagnostic assays for CWD and other prion diseases. Additionally, characterization of the complement of peripherally circulating miRNAs that are associated with the development of prion disease may provide important information to decipher pathophysiological processes in the brain.

## Materials and Methods

### Ethics statement

All procedures involving live hamsters were approved by the Canadian Science Centre for Human and Animal Health - Animal Care Committee (CSCHAH-ACC) according to the guidelines set by the Canadian Council on Animal Care. All protocols were designed to minimize animal discomfort. The approval identification for inoculation of hamsters with 263 K scrapie in this study was animal use document H16-021.

### Animals

Peripheral blood serum from ~7,000 elk originating on farms in Saskatchewan at the time of depopulation during an outbreak of CWD from 1996 to 2002 were kindly provided by Dr. A. Balachandran (Canadian Food Inspection Agency, Ottawa, Ontario, Canada). Brain samples for CWD testing were collected from all animals approximately >1 y of age. IHC staining for PrP^Sc^ to identify CWD positive animals was performed on the medulla oblongata region of the brain at the level of the obex and scored as described^10,36^.

Syrian hamsters were intracranially inoculated using either 263 K scrapie-infected brain homogenate (n = 6) or PBS control brain homogenate (n = 6). Hamster serum collected at 90 days post-infection (dpi) was further processed for NGS.

### PMCA detection of PrP^Sc^ in elk serum

Processing and PMCA reaction of 51 serum samples from elk were carried out as previously described^19^. Duplicates of 225 µL each of serum (except Elk E011, which was processed as a single replicate due to a lack of available volume) were mixed and incubated with 1 volume of 20% (w/v) sarkosyl with gentle inversion for 1 hour at room temperature. Subsequently, samples were centrifuged at 100,000 *x g* for 1 hour at 4 °C after which the supernatant was discarded and the resulting pellet washed with 450 µL 1X PBS at 100,000 *x g* for 30 min at 4 °C. After aspiration of the supernatant, the remaining pellet was directly suspended in 10% brain homogenate from transgenic mice (Tg5037) expressing elk (*Cervus canadensis)* PrP^C^ kindly provided by Dr. Glenn Telling (Colarado State University).

The brain homogenate was prepared in conversion buffer (PBS supplemented with 150 mM NaCl and 1% Triton X-100) with protease inhibitors (Complete EDTA-free, Roche) and further supplemented with 0.025% Digitonin (Invitrogen #BN20061) and 5 mM EDTA (Promega Cat V4231) immediately prior to use. The suspended samples were analyzed in 0.2 ml tubes (Eppendorf, cat. N. 951010022) containing 3 teflon beads (Hoover precision products) and subjected to 3 rounds of PMCA consisting of 144, 96, and 96 cycles, respectively. Each cycle consisted of a 29 min and 40 s incubation at 37 °C, followed by 20 s sonication using an Osonica microsonicator (Model Q700) equipped with a titanium horn. Successive rounds were performed by diluting an aliquot of the preceding round 10-fold in fresh 10% Tg5037 brain homogenate.

### Western blot

Aliquots were taken at the end of each PMCA round and incubated with proteinase K at a concentration of 100 µg/ml with shaking (450 rpm) for 1 hour at 37 °C. The resulting protein was then separated by SDS-PAGE via 12% BT gels (Invitrogen). Duplicates were loaded alongside one another. After electroblotting onto nitrocellulose membrane, the membranes were blocked with 2% (w/v) non-fat milk for 1 hour. Membranes were probed with the anti-PrP primary antibody PRC1 kindly provided by Dr. Glen Telling (1:5000) followed by a sheep anti-mouse secondary antibody (1:3000). Immunoreactive bands were visualized via chemoluminescence assay.

### Elk Genotyping

Buffy coat was isolated from 96 CWD-positive and 95 CWD-negative elk using a Ficoll-Paque gradient. DNA was extracted from the buffy coat as follows: 100–200 μl of buffy coat was incubated at 60 °C overnight in a solution of Proteinase K (80 μl) and tissue lysis buffer (450 μl). Each sample was then extracted three times with phenol, followed by two extractions with 24:1 chloroform/isoamyl alcohol. DNA was precipitated out of the aqueous layer using 1/10^th^ volume of 3 M sodium acetate and 2x volume of 100% ethanol. The pellet was washed with 70% ethanol, then rehydrated with 100 µl of Tris-EDTA buffer.

A 919 bp product spanning the *prnp* coding region was amplified from each elk sample in a 100 μl reaction using the following conditions (final concentration in brackets): 2.5 U Taq polymerase (Invitrogen), 10x buffer (1×), 25 mM dNTPs (2 mM), 50 mM MgCl_2_ (2.5 mM), 5 μM forward primer (0.5 μM), 5 μM reverse primer (0.5 μM), 100 ng of DNA (1 ng/μl). The PCR conditions were as follows: 5 minute denaturing phase at 94 °C, followed by 36 cycles of: 1 minute denaturing at 94 °C; 30 seconds annealing at 64.5 °C; 1 minute extension at 72 °C; with a final extension step of 72 °C for 10 minutes. Sequencing was performed by the DNA Core Facility of the National Microbiology Laboratory using an ABI 3730 DNA Analyzer, and Applied Biosystems BigDye Terminator Version 3.1 chemistry. There are 6 primers that span the 771 bp coding region of the elk PRNP gene (CERMF, gtggaacaagcccagtaaac; CERMR1, gacacagtcatgcacaaagg; CERMup, atgggcatatgatgctgaca; MooseR, gcaagaaatgagacaccacc; reinbouF, aagccacataggcagctgga; reinbouR, ggatcacacttgcccctctt). Sequences were aligned and analyzed, against the reference sequence AY237542, using two software programs *Seqman* (Laser Gene) and *SeqScape v2.5* (Applied Biosystems) to confirm accurate homozygote and heterozygote base-calling.

### Serum miRNA next-generation sequencing

RNA was extracted from serum using the Norgen Biotek Corp. plasma/serum RNA purification mini kit. Sequencing libraries were prepared from RNA using the Illumina TruSeq small RNA library preparation kit according to manufacturer’s instructions with the following modifications. Fifteen cycles were used for the final library PCR amplification step. The sequencing libraries were purified with the BluePippin DNA size selection system by Sage Science using the 3% agarose gel cassettes, gating from 125–150 bp. Prior to sequencing, library purification was confirmed by running samples on an Agilent Bioanalyzer using high-sensitivity DNA chips. Purified libraries were sequenced in multiplex using either the Illumina MiSeq or NextSeq platforms. Samples were multiplexed such that approximately 5 million single-ended 80-nt reads were sequenced for each sample.

### MiRNA sequencing analysis pipeline

Preprocessing was performed using the Galaxy platform. First, adapter sequences were trimmed from the raw small RNA reads (80 nt in length) using Cutadapt, discarding any reads that do not contain the adapter sequences^37^. Next, Trimmomatic was used to trim low quality sequences from the reads using sliding window trimming, dropping reads with an average quality score below 20 and read length below 18^38^. Contaminant reads were removed from the cleaned fastq files by aligning to the UniVec vector contaminant database and then human rRNA reference sequences using Bowtie2 with the default settings^39^. The unaligned reads were written to a fastq file for subsequent alignment to the reference genome. Bowtie2 with the built-in “Very sensitive end-to-end” setting was used to align the cleaned reads to the human GRCh38 and mouse GRCm38 reference genomes. The aligned reads were mapped to known human miRNAs and counted using FeatureCounts with the “forward-stranded” protocol, allowing multi-aligned reads to be mapped^40^. The human and mouse miRNA annotations were downloaded from miRBase in GTF format, and served as the reference file for counting the miRNAs^41^. The human miRNA annotation, which includes just over 2500 miRNAs, was used for subsequent analysis^42^. Further analysis of the miRNA expression data was conducted in RStudio. Read counts were normalized using the DESeq2 package according to a negative binomial model of fitted mean and gene-specific dispersion^43^. Log2-transformed normalized read counts were obtained using the rlog function of DESeq2.

### Serum miRNA species comparison

The miRNA populations of two reference human serum samples and 16 mouse serum samples were sequenced and analyzed identically to the elk and hamster datasets. MiRNA expression datasets were constructed for human and mouse serum using the human and mouse reference annotation information. Expression profiles for miRNA common between elk, hamsters, mice and humans were generated by plotting the mean log2-transformed normalized read count from DESeq2 for each miRNA.

### Biomarker identification

Hemolyzed elk serum samples were identified based on a read count ratio between miR-451a and miR-23a-3p greater than 5^44^. DESeq2 was used for read count normalization and differential expression analysis between CWD-positive and -negative elk serum samples in the full dataset (35 CWD (−) and 35 CWD (+) samples), and the cleaned dataset (21 CWD (−) and 15 CWD (+) samples) with hemolyzed samples removed^43^. Differential expression analysis was also performed between the six 263 K scrapie-infected hamsters and six PBS mock-infected hamsters using DESeq2. P-values for each miRNA were FDR corrected using the Bonferonni-Hochberg method. Significantly altered miRNAs were identified based on fold change <−1.5 or >1.5 and FDR corrected p-value <0.05. The R package Pheatmap was used for hierarchical clustering of significantly altered miRNAs and visualization of the relative log2-transformed read counts as heatmaps^45^. The log2-transformed normalized miRNA read counts from DESeq2 were also supplied for principal component analysis using the DESeq2 package^43^.

### Logistic regression

The full 70 elk serum samples were classified based on CWD status assigned by obex IHC (1 = CWD positive, 0 = CWD negative). The log2-transformed normalized read counts of candidate serum miRNA biomarkers in each sample were supplied as dependent variables to construct lasso penalized logistic regression models using the glmnet package^46^. Ten-fold cross-validation was used to compute the optimal lambda shrinkage coefficient that minimizes cross-validated error and the largest value of lambda within one standard error of this optimal value. These lambda values were then supplied to construct penalized logistic regression models. The diagnostic potential of the logistic regression models were assessed using receiver operating characteristic (ROC) analysis with the ROCR package^47^. The ROCR package was also used for ROC analysis of normalized read count for each individual miRNA biomarker to discriminate CWD positive from negative elk serum samples. The area under the curve (AUC) for ROC analysis was used to compare the discriminative capabilities for miRNAs and logistic regression models. Logistic regression models were further validated using repeated cross-validation by randomly splitting the 70 elk serum samples into 56 samples for training and 14 samples for testing and reporting the ROC AUC. This was repeated 500 times and the 95% confidence interval (CI) was reported for AUC.

### miRNA Target identification and functional annotation

DNA Intelligent Analysis (DIANA)-miRPath v3.0 was used to identify KEGG molecular pathways that are potentially impacted by alterations in abundance of a subset of miRNAs^48–51^. Analysis was performed using the algorithm to link miRNAs to target genes annotated in Tarbase, v7.0 using the ‘pathways union’ option. P-values were obtained by Fisher’s exact test (hypergeometric distribution) as enrichment analysis method and the false discovery rate (FDR) correction (Benjamini and Hochberg) using an FDR <0.001 as the significance threshold.

Target genes of the group of 6 miRNA biomarkers common to elk and hamster were identified by comparing four databases: miRDB^52^, Targetscan^53^, miRWalk^54^ and Tarbase v8^55^. Genes common to all four databases were reported as targets for each miRNA. A miRNA-target interaction network was constructed using Cytoscape^56^. Gene set enrichment analysis was performed on target genes using Enrichr^57,58^. Enriched gene sets were ranked based on adjusted p-value for the Reactome 2016 database and gene ontologies for molecular function, biological process and cellular compartment.

## Results

### Study population

**Figure 1:**
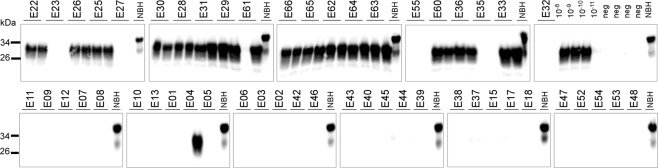
PMCA of CWD PrP^Sc^. PrP^Sc^ detection in serum of 51 elk, 21 of which were CWD IHC positive (top) and 30 IHC negative (bottom). Samples were subjected to three serial rounds of PMCA and the results of the third round are shown. Samples were treated with proteinase K except for the normal brain homogenate (NBH) used as a migration control. Numbers on the left indicate molecular weight in kDa.

Peripheral blood sera originating from ~7,000 elk on farms in Saskatchewan, Canada depopulated during outbreaks of chronic wasting disease from 1996 to 2002 were used as source material for this study^36^. Of these, 231 were from animals that tested positive for CWD by IHC staining of obex tissue for PrP^Sc^ by the Canadian Food Inspection Agency’s CWD national reference laboratory. In total, 70 of these samples were used in this study, 35 from elk that tested positive by IHC and 35 from IHC-negative animals. In the current study, information was not available regarding which animals exhibited clinical signs at the time of euthanasia, therefore sera from IHC-positive animals were assumed to include both clinical and preclinical cases of CWD infection. IHC scoring were available as described in a 2005 publication from Spraker *et al*. for all 35 positive elk on a scale of 1–4^10^. No animals scored 4 and so it is likely that the sampled elk were all at the preclinical stage of disease. The presence or absence of PrP^Sc^ in blood was confirmed in 51 of these serum samples using the PMCA assay. Of the 21 sera from IHC-positive elk, 16 were positive by PMCA, while 1 of the 30 IHC-negative elk gave a positive PMCA result (Fig. 1).

### Elk serum miRNA profiling using Illumina NGS

**Figure 2:**
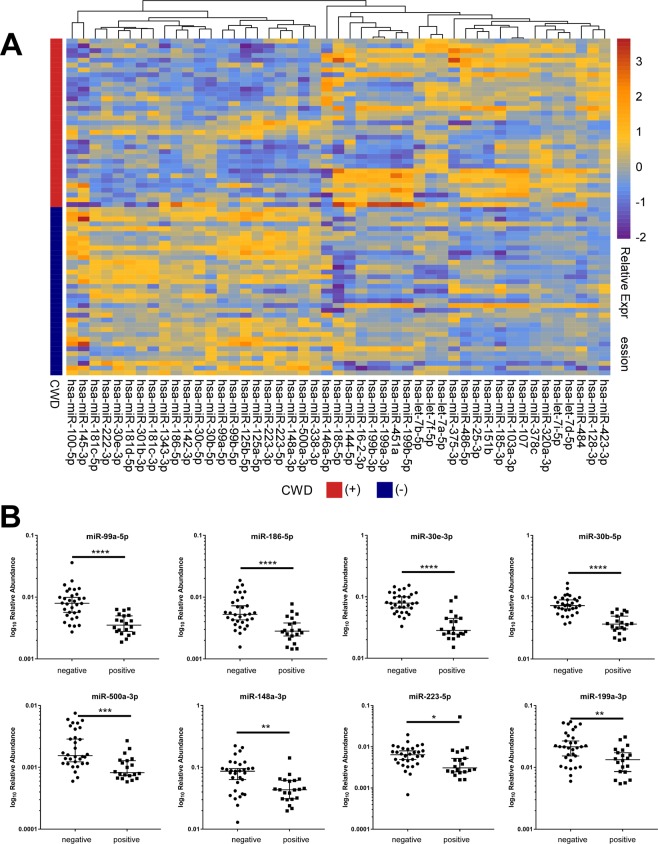
Differentially expressed miRNA in serum from CWD infected elk. (**A**) Hierarchical clustering using relative log transformed read count of 47 differentially expressed miRNAs reveals an alternate pattern of abundance in serum taken from elk that were CWD positive by IHC. (**B**) Increases of 8 miRNAs in CWD positive elk confirmed by qRT-PCR. Serum miRNA levels were reported as relative miRNA concentration, with the formula 2^−ΔCt^ normalized to miR-23a-3p. The log10 relative abundance was plotted and significant median differences in the miRNA levels between each group determined by the nonparametric Mann-Whitney test (*p <0.05, **p <0.01, ***p <0.001, ****p <0.0001). The levels of miR-423-3p, miR-486-5p, miR125a-5p and miR-30-5p were unchanged.

RNA isolated from the sera of 35 IHC-positive elk and 35 IHC-negative animals were sequenced using Illumina NGS to determine the differential miRNA profiles of infected and uninfected animals. As there is no available reference genome and accompanying miRNA annotation for elk, we used human and mouse reference sequences to identify conserved miRNAs in elk serum. The abundances of 367 miRNAs that were identified using either the human (GRCh38) or mouse (GRCm38) miRNA reference annotation were almost identical, and so the human reference genome (GRCh38) and associated miRBase annotation were used (Supplementary Fig. 1). In total 593 miRNAs were identified in elk serum, of which 47 showed altered expression in IHC-positive elk compared with IHC-negative animals. Of these 47 miRNAs, 25 had increased abundance in IHC-positive elk (>1.5-fold, FDR-corrected p-value <0.05) and 22 had decreased abundance (<−1.5-fold, FDR-corrected p-value <0.05) (Fig. 2A). Quantitative RT-PCR was used to confirm the relative abundance of 12 of the 47 altered miRNAs. MiRNA levels were normalized to that of miR-23a, which was selected by the NormFinder algorithm as the miRNA with the most stable expression across all of the serum samples^59^. The results are shown in Fig. 2B. Of the 12 miRNAs chosen, 8 also showed altered abundance in IHC-positive animals by RT-PCR (p <0.05).

**Figure 3:**
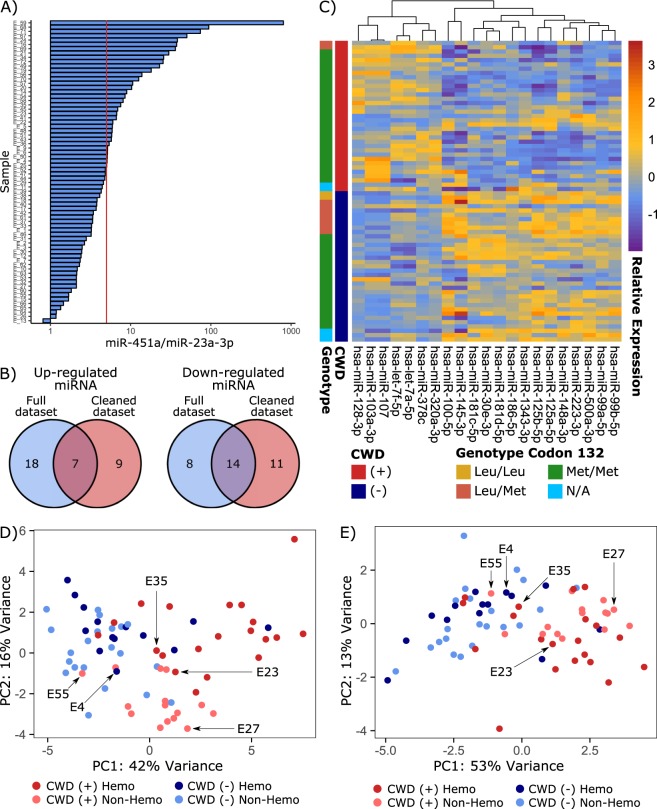
Serum miRNA are biomarkers of CWD in elk. A subset of serum miRNAs are altered in CWD infected elk regardless of the presence of hemolysis in serum. (**A**) A cleaned elk dataset was constructed by removing hemolyzed samples using a strict miR-451a/miR-23a-3p cutoff of 5. (**B**) 21 miRNAs were detected as differentially expressed regardless of the presence of hemolyzed serum samples in the dataset. (**C**) Hierarchical clustering of the putative 21 serum miRNA biomarkers highlights their altered abundance elk positive for CWD by IHC, and their expression is not influenced by the genotype at codon 132 of PrP. (**D**) Elk serum samples cluster based on both disease status and presence of hemolysis when the list of genes altered in the full dataset is used for PCA. (**E**) Elk serum samples cluster based on disease status, but not hemolysis, when the 21 putative CWD biomarkers are used for PCA. The IHC (−) elk that was (+) by PMCA (E4) and the four IHC (+) elk that were (−) by PMCA are highlighted on the PCA plots.

Some miRNAs are highly enriched in red blood cells, and it is well known that hemolysis can interfere in the analysis of miRNA abundance from serum and plasma samples^60,61^. A number of the elk serum samples used varied visibly in color with some appearing pink indicating that some level of hemolysis may have occurred. This was unsurprising, as blood samples were collected in a field rather than experimental setting. As it is important in biomarker discovery in sera to minimize the effect of variable amounts of miRNAs originating from cells, we assessed hemolysis in each sample by determining the relative levels of miR-451, a miRNA highly expressed in red blood cells, and miR-23a, which is unaffected by hemolysis. Serum samples were considered unaffected by hemolysis if by RT-PCR the delta Cq between these 2 miRNAs was less than 5^44^. This criterion, which was considered stringent, reduced the dataset to 15 IHC-positive and 21 IHC-negative serum samples in which hemolysis was unlikely to interfere with miRNA profiling (Fig. 3A). These data were reanalyzed and in this case 41 miRNAs had altered abundance in elk that were positive by IHC compared with animals that tested negative (<−1.5 or >1.5 and FDR corrected p-value <0.05. Of these, 21 miRNAs were also found in the 47-miRNA signature identified in the analysis of all 70 elk (Fig. 3B). Figure 3C displays a heatmap of the abundance of these 21 serum miRNAs that were unaffected by hemolysis in all 70 elk.

North American elk are polymorphic for two common alleles of the prion gene (*PRNP*), encoding either methionine (M132) or leucine (L132) at codon 132. The genotype at codon 132 appears to influence CWD susceptibility and disease expression in elk, with homozygosity or heterozygosity for leucine associated with longer incubation periods, as well as differing biochemical properties of PrP^Sc ^^62,63^. We determined the codon 132 genotypes of 65 of the 70 elk included in our study and identified the L132 allele in 12 animals, of which 10 were IHC-negative. This genotype-phenotype association was expected, as it is well known that the L132 allele confers some resistance to CWD. However, we found no relationship was apparent between the codon 132 genotype and the disease-related miRNA profile (Fig. 3C).

Hierarchical clustering of the miRNA patterns was performed, using principal component analysis (PCA) to represent the overall expression pattern of the 47-miRNA subset in all 70 serum samples (Fig. 3D). Whilst the IHC-positive and -negative samples cluster together for the most part, the most highly hemolyzed samples also form clear clusters (Fig. 3D). Using the 21-miRNA subset established after taking hemolysis into account, the separation of IHC-positive and -negative elk is more clearly evident (Fig. 3E). These results indicate that the signature of 21 differentially abundant serum miRNAs in CWD-infected elk is unlikely to be strongly influenced by hemolysis, and is more likely to be an indicator of disease. The single IHC-negative elk from which serum was positive by PMCA (E4) as well as the four IHC-positive elk from which serum was negative via PMCA (E23, E27, E35 and E55) are highlighted on the PCA plots (Fig. 3D,E). E23 and E27 cluster with the IHC-positive samples in PCA, while E55 and E35 cluster with the IHC-negative samples. It is possible that E35 and E55 were pre-clinical animals that tested positive via IHC, whereas E23 and E27 were in the later stages of disease, but had lower prion titres in peripheral tissues and thus tested negative via PMCA. E4 clusters with the IHC-negative samples despite being detected as positive via PMCA.

We also analyzed the data in relation to the IHC scoring annotation that were available for these elk; all had scores of 1, 2 or 3 on a scale of 1–4. These scores did not correlate with the results of PMCA. Also the samples did not appear to cluster based on IHC scores and we provide this data as Supplementary Fig. 3^10^. Unfortunately, of the 6 animals with the earliest detectable disease score of 1, four were amongst the most hemolyzed samples and we were not confident in using scores to group the animals for further statistical analysis. We concluded that in this sample set biological variation is greater than any differences in miRNA abundance that correlate with IHC scores.

### Diagnostic potential of serum miRNA biomarkers

**Figure 4:**
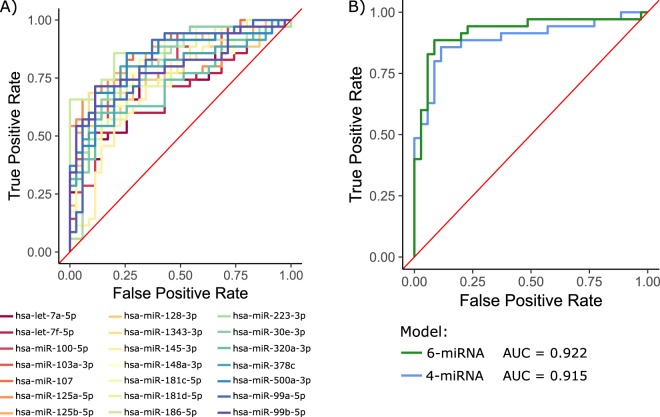
Diagnostic potential of serum miRNA biomarkers. The ability for the relative abundance of each miRNA biomarker in serum to differentiate CWD positive from negative elk (by IHC) was visualized using ROC analysis. (**A**) ROC curves were constructed for each of the 21 putative serum miRNAs of CWD in elk. Logistic regression was used to model CWD status (classified as 1 = CWD positive, 0 = CWD negative; CWD status was assigned by IHC) with abundance of serum miRNA biomarkers. Lasso penalized logistic regression models were built using the log2 transformed read counts of 6 (hsa-miR-185-5p, hsa-miR-500a-3p, hsa-miR-99a-5p, hsa-miR-125b-5p, hsa-miR-181c-5p and hsa-miR-181d-5p) and 4 (hsa-miR-30e-3p, hsa-miR-500a-3p, hsa-miR-125b-5p, hsa-miR-181c-5p) miRNAs. (**B**) The diagnostic accuracy for each logistic regression model was assessed using ROC analysis.

To assess the capabilities of miRNAs to discriminate between serum samples from IHC-positive and –negative elk, receiver operating characteristic (ROC) analysis was applied to the log2 transformed normalized read count of each of the putative biomarkers across the 70 elk samples. ROC curves were plotted for each of the 21 miRNAs individually (Fig. 4A). The area under the curve (AUC) for a ROC curve is equal to the probability of a randomly drawn disease-positive sample having a higher value of a test result than a randomly drawn disease-negative sample. The AUCs of the miRNAs ranged from 0.70 (hsa-let-7f-5p) up to 0.86 (hsa-miR-186-5p). Ten of the 21 miRNAs had AUCs >0.8, indicating good diagnostic discrimination.

We conjectured that CWD status may be predicted more accurately jointly by the altered abundances of multiple miRNA biomarkers. Towards this end, logistic regression was used to model IHC status with the abundance of individual miRNA biomarkers in elk serum as mutually independent predictor variables. The full 70 elk serum samples were classified based on IHC status (1 = positive, 0 = negative) and used to build lasso penalized logistic regression models with the log2 transformed read count values of the 21 miRNAs. Lasso penalized logistic regression uses a coefficient shrinkage constant (known as lambda) to force the coefficients of less contributive predictors to zero, thereby reducing the risk of overfitting. We used k-fold cross-validation to determine the value of lambda that minimizes the mean cross-validated error. This reduced the predictors in the logistic regression model to 6 miRNAs (hsa-miR-185-5p, hsa-miR-500a-3p, hsa-miR-99a-5p, hsa-miR-125b-5p, hsa-miR-181c-5p and hsa-miR-181d-5p). To limit the complexity of the logistic regression model, we also used k-fold cross-validation to find the value of lambda such that the error is within one standard error of the minimum mean error. This reduced the number of predictors to 4 miRNAs (hsa-miR-30e-3p, hsa-miR-500a-3p, hsa-miR-125b-5p, hsa-miR-181c-5p). Using a prediction probability cutoff of 0.5, the 6-miRNA model incorrectly assigned CWD status to 10/70 elk samples while the 4-miRNA model incorrectly assigned 11/70 samples.

ROC analysis was used to compare the discriminative accuracies of the 2 logistic regression models of CWD status. ROC curves for the models built with 6 and 4 miRNAs yielded AUC values of 0.922 and 0.915 respectively (Fig. 4B). We further validated the two models using repeated cross-validation repeated 500 times. This resulted in AUC values of 0.851 (95% CI = 0.844, 0.858) and 0.793 (95% CI = 0.785, 0.801) respectively for the 6- and 4-miRNA models. These results suggest that logistic regression models based on serum miRNA expression biomarkers in elk can be used to predict CWD status. Models built using a reduced number of biomarkers have reduced discrimination; however they are still able to correctly discriminate the majority of elk that tested positive by IHC.

### A subset of serum miRNAs can be used to discriminate prion infection in hamsters and elk

**Figure 5:**
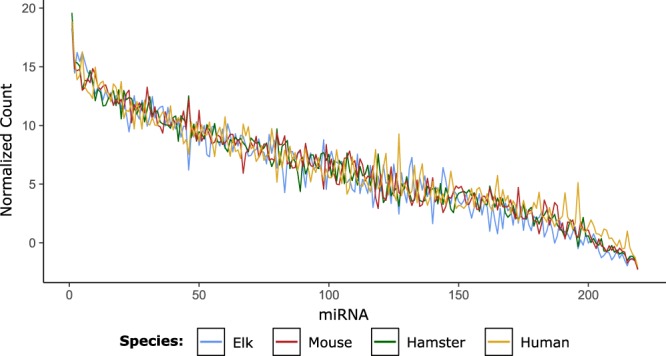
Serum miRNA abundance is conserved between elk, hamsters, mice and humans. The mean log2 transformed normalized read count was plotted for 219 miRNAs commonly detected in elk, hamster, mouse and human serum.

As we restricted our analysis to miRNAs broadly conserved amongst mammals, we were interested to determine whether the miRNA signature we identified for CWD in elk was associated with prion disease in other species. We therefore profiled miRNAs from the sera of 6 hamsters infected with the 263 K strain of scrapie and 6 mock-infected hamsters. The animals were sacrificed at 90 days post-infection when clinical signs were evident, and peripheral blood was collected. In total, 426 miRNAs were identified in hamster serum, again using the human reference genome and miRBase to map sequences to known miRNAs. Of note, although hamster and elk samples were prepared for sequencing using the same methodology, hamster serum was found to contain a relatively lower relative proportion of miRNA sequences and a much higher relative proportion of small RNAs that were unmapped (Supplementary Fig. 2). The ratio of miR-451/miR-23a was also higher in hamsters than in elk, although the levels of miR-23a were again very stable between individual animals. This could indicate either greater levels of hemolysis in the hamster serum or a relatively lower proportion of miR-23a present in circulating RNA in hamsters. To ensure the relative abundances of conserved miRNAs were similar between species we plotted the normalized read counts of 219 abundant conserved miRNAs from elk and hamsters, as well as from samples of mouse and human serum prepared in our laboratory. Overall, the relative abundance/expression profiles of these miRNAs were strikingly similar between species (Fig. 5).

**Figure 6:**
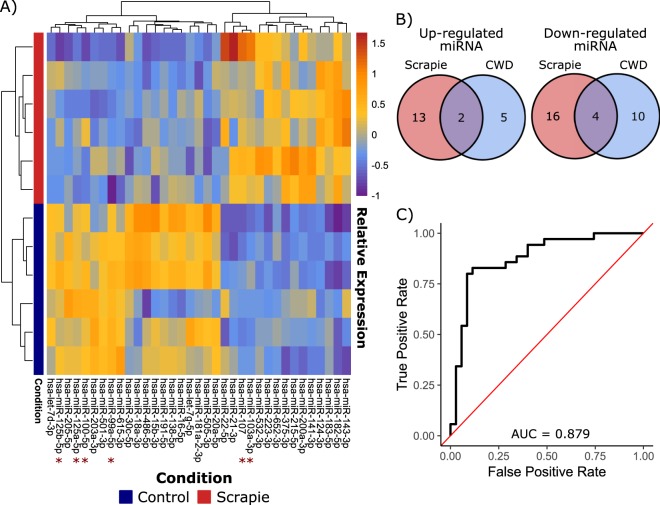
Serum miRNA are also biomarkers of prion infection in scrapie-infected hamsters. A subset of serum miRNA biomarkers of CWD in elk showed similar alterations in abundance in scrapie-infected hamsters. (**A**) Scrapie-infected hamster samples cluster together based on expression of serum miRNA biomarkers. The 6 biomarkers common to CWD positive elk and scrapie-infected hamsters are highlighted by a red asterisk (*). (**B**) A signature of 6 altered serum miRNAs is common to CWD-infected elk and scrapie-infected hamsters. (**C**) Lasso penalized logistic regression was used to model elk CWD status using the expression of 5 of the 6 conserved miRNAs (hsa-miR-103a-3p, hsa-miR-107, hsa-miR-99a-5p, hsa-miR-125b-5p and hsa-miR-100-5p) in 70 elk serum samples. ROC analysis revealed that this model was able to correctly identify CWD status in the majority of elk samples.

Differential expression analysis was performed on the hamster dataset and a total of 35 serum miRNAs were identified that differed in abundance between 263 K scrapie infected and uninfected hamsters (−1.5< FC >1.5, FDR corrected p-value <0.05). The alternate pattern of abundance displayed by these miRNAs in scrapie-infected hamsters was visualized as a clustered heatmap (Fig. 6A). A total of 10 miRNAs had differential abundance in both scrapie-infected hamsters and IHC-positive elk (hsa-miR-99a-5p, hsa-miR-375-3p, hsa-miR-223-3p, hsa-miR-107, hsa-miR-125a-5p, hsa-miR-103a-3p, hsa-miR-100-5p, hsa-miR-30c-5p, hsa-miR-486-5p, and hsa-miR-125b-5p). This list of 35 altered miRNAs was compared to the 21 biomarkers identified in CWD-infected elk, and 6 were found in common (Fig. 6B). Two of these 6 miRNAs (miR-103a-3p and miR-107) showed higher abundances in diseased individuals of both species, while 4 (miR-125a-5p, miR-125b-5p, miR-100-5p and miR-99a-5p) were less abundant in diseased individuals. Interestingly, these miRNAs take on similar patterns of expression in both elk and hamster serum. MiR-103a-3p and miR-107 form a distinct cluster across both the elk and hamster samples (Figs. 3C and 6A). This is also true for miR-125a-5p and miR-125b-5p.

The 6 commonly altered miRNAs were among the best candidate CWD biomarkers; 5/6 miRNAs had an AUC >0.8 when ROC analysis was applied to these miRNAs individually with the 70 elk samples. To further assess the predictive power of this 6-miRNA signature we again used lasso logistic regression to predictively model IHC status in the 70 elk samples. In this case, using the lambda value that minimized cross-validated error reduced the predictors to 5 miRNAs (hsa-miR-103a-3p, hsa-miR-107, hsa-miR-99a-5p, hsa-miR-125b-5p and hsa-miR-100-5p). Using the same prediction probability cut-off of 0.5, this model incorrectly assigned IHC status to 10 of the 70 elk (4 false positives and 6 false negatives). ROC analysis was used to assess the diagnostic accuracy of this model, giving an AUC of 0.879 (Fig. 6C). Repeated cross-validation was also used to get a better estimate of the discriminative accuracy of this 5-miRNA model, resulting in an AUC of 0.867 (95% CI = 0.859, 0.872). Therefore, the logistic regression model built using these 5 commonly altered miRNAs can be used to discriminate CWD status with similar accuracy to the other models investigated here.

### Functional annotation of serum miRNA biomarkers

To infer the biological function that may be related to the development of CWD, we first used the DIANA miRPath v3.0 tool to identify 20 enriched pathways (adjusted P-values <0.001) targeted by the 21 miRNAs that showed altered abundance in CWD positive elk and were not affected by hemolysis^48^. The top 15 pathways are reported in Table 1. Notably, the top scoring pathway was the KEGG pathway relating to the development of prion diseases that was directly targeted by miR-148a-3p, miR-186-5p, miR-30e-3p, all three of which were decreased in abundance in sera from CWD positive animals. Functionally, a decrease in abundance of a regulatory miRNA would imply that its targets would be increased in abundance. Predicted targets were taken from DIANA-TarBase v.07^48^ that includes miRNA interactions predicted from HITS-Clip and other experimentally derived platforms. Notably, all three of these miRNAs have been shown to target the PRNP gene itself. The other genes targeted in this pathway were STIP1, HSPA1A, IL1A and MAPK1 (Supplementary Fig. 4). In addition, pathways involved in fatty acid metabolism and biosynthesis lysine degradation, ECM-receptor interaction, TGF-beta signaling and carcinogenesis were also found to be significantly enriched.

**Table 1:** KEGG pathways involving the 21 miRNAs altered in abundance in sera of CWD positive elk.

Rank	KEGG Pathways	Adjusted *P*-value	Genes	MiRNA
1	Prion diseases	<1 × 10^−325^	5	3
2	ECM-receptor interaction	<1 × 10^−325^	23	4
3	Fatty acid metabolism	<1 × 10^−325^	16	5
4	Fatty acid biosynthesis	<1 × 10^−325^	5	6
5	Hippo signaling pathway	<1 × 10^−325^	76	8
6	Viral carcinogenesis	<1 × 10^−325^	125	10
7	Proteoglycans in cancer	<1 × 10^−325^	121	12
8	Lysine degradation	<1.78 × 10^−15^	31	9
9	Cell cycle	<6.55 × 10^−15^	82	9
10	Hepatitis B	<7.44 × 10^−11^	75	7
11	Chronic myeloid leukemia	<9.1 × 10^−11^	44	9
12	Adherens junction	<2.1 × 10^−10^	46	9
13	Glioma	<8.34 × 10^−10^	37	9
14	P53 signaling pathway	<3.91 × 10^−9^	44	8
15	TGF-beta signaling pathway	<5.89 × 10^−7^	47	6

KEGG molecular pathways that were enriched with miRNA target genes were identified using DNA Intelligent Analysis (DIANA)-miRPath v3.0^48–51^.

**Figure 7:**
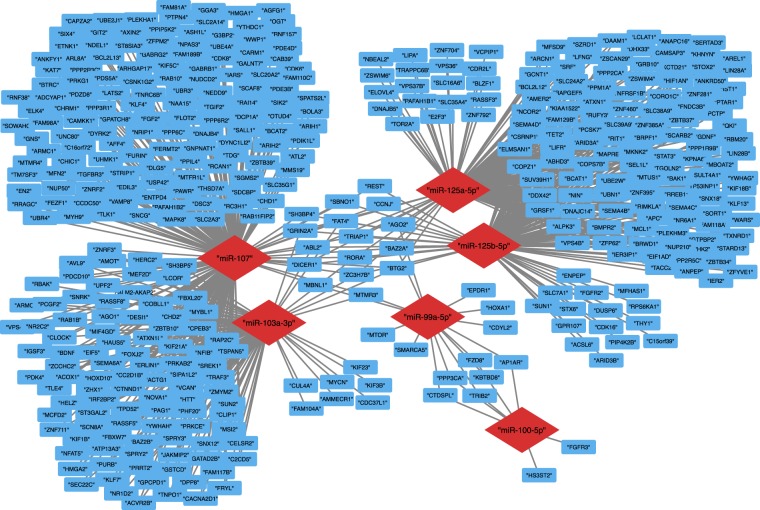
Interaction network of 6 altered serum miRNA in prion-infected elk and hamster with target genes. The conserved 6-miRNA signature targeted a total of 422 target mRNA molecules. Red diamonds indicate miRNAs and blue rectangles indicate target genes.

We further investigated the target genes of the 6 miRNA signature that was determined to be common to prion infection in elk and hamsters. Targets were predicted using 4 different databases; miRDB^52^, Targetscan^53^, miRWalk^54^ and Tarbase v8^55^. For each miRNA, genes common between all four databases were assigned as targets. This resulted in a total of 422 predicted targets for which a miRNA-target interaction network was constructed using the Cytoscape tool (Fig. 7). The 6 miRNAs formed 3 distinct clusters based on their shared target genes; miR-107 and miR-103a-3p, miR-125a-5p and miR-125b-5p, and miR-99a-5p and miR-100-5p each form a distinct 2-miRNA cluster. Many target genes are shared by the miRNAs within each cluster as they are highly related (Table 2)^42^. The miRNAs in the miR-103a/107 and miR-99a/100 clusters differ only by a single nucleotide, while miR-125a has a two nucleotide insertion compared to miR-125b-5p. Relatively few target genes were common between the three miRNA clusters. The Enrichr gene set enrichment tool was used to infer their biological function^57,58^. Enriched gene sets were ranked based on adjusted p-value, and the top 5 entries for the Reactome 2016 database, as well as gene ontologies (GO) for biological process, molecular function and cellular compartment are reported in Table 3. Many of the enriched Reactome 2016 gene sets were related to membrane trafficking and vesicle mediated transport, as well as regulation by TP53. Many of the enriched GO biological process and molecular function gene sets were related to transcriptional regulation and ubiquitin ligase activity.

**Table 2:** Clustered miRNAs share highly similar mature sequences.

miRNA	Mature Sequence	Base Mean Count
hsa-miR-103a-3p	agcagcauuguacagggcuauga	12050
hsa-miR-107	agcagcauuguacagggcuauca	5911
hsa-miR-99a-5p	aacccguagauccgaucuugug	128
hsa-miR-100-5p	aacccguagauccgaacuugug	176
hsa-miR-125a-5p	ucccugagacccuuuaaccuguga	261
hsa-miR-125b-5p	ucccugagacccuaacuuguga	56

**Table 3:** Functional annotation of miRNA target genes.

Name	Adjusted p-value	z-score	Combined score
**Reactome 2016**			
Membrane Trafficking_Homo sapiens_R-HSA-199991	1.27E-06	−2.15	43.33
Vesicle-mediated transport_Homo sapiens_R-HSA-5653656	2.52E-05	−2.16	35.60
Golgi-to-ER retrograde transport_Homo sapiens_R-HSA-8856688	8.97E-04	−1.95	24.40
Transcriptional Regulation by TP53_Homo sapiens_R-HSA-3700989	3.26E-03	−2.28	24.97
Intra-Golgi and retrograde Golgi-to-ER traffic_Homo sapiens_R-HSA-6811442	4.16E-03	−1.96	20.58
**GO Biological Process**			
regulation of transcription from RNA polymerase II promoter (GO:0006357)	2.99E-07	−1.38	31.37
positive regulation of transcription, DNA-templated (GO:0045893)	7.40E-07	−1.71	36.29
positive regulation of transcription from RNA polymerase II promoter (GO:0045944)	8.41E-05	−1.90	30.38
ubiquitin-dependent protein catabolic process (GO:0006511)	6.62E-04	−1.08	14.71
regulation of transcription, DNA-templated (GO:0006355)	1.48E-03	−1.83	22.51
**GO Molecular Function**			
transcriptional activator activity, RNA polymerase II transcription regulatory region sequence-specific binding (GO:0001228)	2.58E-04	−1.46	20.52
core promoter binding (GO:0001047)	2.58E-04	−1.28	17.57
ubiquitin protein ligase activity (GO:0061630)	5.63E-04	−1.69	21.24
transcription regulatory region sequence-specific DNA binding (GO:0000976)	5.63E-04	−1.09	13.42
ubiquitin-like protein ligase activity (GO:0061659)	1.06E-03	−1.90	21.75
**GO Cellular Component**			
cytoplasmic vesicle membrane (GO:0030659)	4.10E-03	−1.56	17.03
kinesin complex (GO:0005871)	8.28E-03	−1.97	18.29
fibrillar center (GO:0001650)	8.28E-03	−1.69	15.34
micro-ribonucleoprotein complex (GO:0035068)	2.11E-02	−3.16	24.10
nucleolar part (GO:0044452)	2.11E-02	−1.35	10.44

Enriched gene sets were identified using Enrichr^57,58^. GO: gene ontology.

## Discussion

Circulating miRNAs are promising biomarkers for clinical diagnosis and prognosis or therapeutic trials. This is the first study, to our knowledge, to determine global alterations in circulating miRNAs in animals naturally infected with a prion disease.

We used NGS to identify 47 miRNAs altered in the serum of elk that tested positive for CWD. Given the lack of available annotation of the elk genome we restricted our study to those mature miRNA sequences that are highly conserved amongst mammals. A number of miRNAs are well known to be present at high levels in red blood cells and their release into serum can be a confounding factor in the identification of reliable disease-related biomarkers. Accordingly, we determined which of the 47 altered miRNAs exhibited abundance that was unaffected by hemolysis, reducing the signature to 21 miRNAs. Statistical analysis revealed that the abundance of these miRNAs could be used to discriminate CWD status in elk as assigned by IHC and PMCA. Of note we also identified 6 of these miRNAs to be altered in the serum of an experimental rodent model of prion disease: 263 K infected hamsters. A logistic regression model built with 5/6 of these miRNAs also exhibited good discriminative accuracy. Therefore there is potential to determine common miRNA biomarkers of prion disease between different animal species and prion strains.

Of the 47 miRNAs, 19 (40%) have previously been determined to have altered abundance in brain tissues from humans and animals with prion diseases^22,24–26^. Of note 7 were identified in more than one study: miR-128-3p, miR-7d-5p, miR-7i-5p, miR-451a, miR-338-3p, miR-125a-5p and miR-146a-5p. This limited overlap is unsurprising as not all miRNAs altered in brain tissue may cross the blood brain barrier and be reliably detected in serum. As we were unable to annotate our data with the elk genome, it is also possible that some differences may be attributed to species-related differences. We did however note that the similarities between the miRNA profile identified in hamster serum and those described in previous studies was higher, 20 out of 35 (57%). This may reflect the fact that the majority of reported studies have been performed in rodent models of prion disease.

A single study has previously reported miRNA alterations in serum^33^. Using targeted qRT-PCR, increased levels of miR-21-5p and miR-342-3p were found to be altered in the serum of sheep naturally affected by scrapie. However, we did not identify these miRNAs in this study. As there were no global studies reporting serum miRNA profiles in prion-infected animals we also compared our data with similar studies on patients suffering from Alzheimer’s disease, which is probably the most extensively studied neurodegenerative disorder, and found a number of candidate miRNA biomarkers in common. These included miR-125b-5p^64–69^, let-7f^70–72^, miR-107^70,73,74^, let-7d-5p, miR-103a-3p^72,75^, miR-146a-5p^67,76^, miR-142-3p^75,77,78^ and miR-181c-3p^79,80^. Notably, 3 of these 7 miRNAs are part of the 6 miRNA cluster similarly altered in hamster serum, strengthening the hypothesis that common indicators of neurodegenerative disease can be determined by analysing the serum profiles of conserved miRNAs. Further literature searches confirmed that in addition to Alzheimer’s Disease, miR-103a and miR-107 have been identified to be altered in the serum of Parkinson’s Disease patients as well as in those suffering from frontotemporal lobar degeneration^81–83^. Downregulation of miR-107 may contribute to Alzheimer’s pathogenesis through deregulation of targets such as granulin, p35, SYK and BACE1^84–87^. Similarly, miR-99a and miR-100 may be regulated by endoplasmic reticulum stress^88,89^ and miR-125a and miR-125b are brain enriched miRNAs involved in the regulation of synaptic plasticity^65,69,90^.

Functional analysis of the 21 miRNAs that showed altered abundance in CWD positive elk and not affected by hemolysis revealed numerous KEGG pathways that were enriched in their putative targets. It was promising that the top scoring pathway was related to the development of prion diseases, with 3 out of the 21 miRNA signature (miR-148a-3p, miR-186-5p, miR-30e-3p) directly targeting the PRNP gene. Their role in prion pathobiology would be an interesting avenue for further study. The 6 miRNAs also altered in hamster serum, as well as serum from patients suffering from other neurodegenerative conditions such as Alzheimer’s and Parkinson’s disease, shared many of the same target genes. Many were particularly related to membrane trafficking and vesicle-mediated transport and the ubiquitin-protease system (UPS). It is possible that these miRNAs contribute to the disruptions in membrane trafficking, which is known to be a feature of prion infection and other neurodegenerative disorders^91,92^. Furthermore, the UPS is known to be impaired during prion infection, potentially contributing to the neurotoxicity that is associated with disease progression^93,94^.

Circulating miRNA signatures, such as those identified in this study, could potentially be used as non-invasive markers to help identify prion infection in elk and other species. However, diagnostic assays based on miRNA expression would be unlikely to provide the same level of accuracy as assays which rely on specific detection of PrP^Sc^ such as IHC, PMCA or RT-QuIC. It is possible that further refinement in the study design could increase specificity. For example, the inclusion of samples from an experimental study where blood was collected at timed intervals post-infection would be useful to tease out those miRNAs that may show altered abundance specifically at early stages of diseases. In addition, a refinement of the IHC scoring system used for grading of naturally infected cervids has recently been described^95^. This introduced a 10 point scale which will undoubtedly be important in accurate annotation of samples for more sophisticated statistical analysis in future studies. In this analysis we know that the CWD positive animals represented a range of stages in disease progression. It is possible that miRNA profiles could identify some animals below the sensitivity of detection of the IHC and PMCA used in defining the CWD positive and negative sample set for this analysis. Nonetheless, we believe that our study has particular strengths of design, namely the inclusion of a greater number of individual animals than is available from experimental studies, a bias-free sampling methodology, a natural route of infection and a reduction in confounding factors such as diet and environment as animals originate from different herds.

## Supplementary information

Supplementary Data

## Data Availability

Data files and R code are available at https://github.com/jslota/CWD_serum_miRNA. Raw data is available on NCBI GEO with the Accession Number GSE13950.
