# Supplementary material for: Identification of circulating microRNA signatures as potential biomarkers in the serum of elk infected with chronic wasting disease
Source: Sci Rep. 2019 Dec 23;9:19705. doi: 10.1038/s41598-019-56249-6 (PMC6928025; doi:10.1038/s41598-019-56249-6)

Identification of circulating microRNA signatures as potential biomarkers in the serum of elk infected with chronic wasting disease

Jessy A. Slota[1,2], Sarah J. Medina[1], Megan Klassen[1], Damian Gorski[4], Christine M. Mesa[1], Catherine Robertson[1], Gordon Mitchell[5], Michael B. Coulthart[3], Sandra Pritzkow[4], Claudio Soto[4] and Stephanie A. Booth[1,2*]

Affiliations

[1]Zoonotic Diseases & Special Pathogens, Public Health Agency of Canada, National Microbiology Laboratory, 1015 Arlington St., Winnipeg, MB R3E 3R2, Canada

[2]Department of Medical Microbiology and Infectious Diseases, Faculty of Health Sciences, University of Manitoba, 730 William Ave., Winnipeg, MB R3E 0W3, Canada

[3]Canadian Creutzfeldt-Jakob Disease Surveillance System, Centre for Foodborne, Environmental and Zoonotic Infectious Diseases, Public Health Agency of Canada, Ottawa, ON, K1A 0K9, Canada

[4]Mitchell Center for Alzheimer's Disease and Related Brain Disorders. Department of Neurology. University of Texas Health Science Center at Houston. 6431 Fannin St. Houston, Texas 77030, USA.

[5]National and OIE Reference Laboratory for Scrapie and CWD, Canadian Food Inspection Agency, Ottawa Laboratory Fallowfield, Ottawa, ON K2H 8P9, Canada

*Corresponding author

E-mail: Stephanie.Booth@canada.ca (SAB)

Tel: (204) 789 6031

Further Information for Figure 1: **PMCA of CWD PrP^{Sc}.**

- Cropped gels were from the 3rd round of PMCA were prepared for Figure 1 (below).
- Each panel represents a single gel/Western blot.
- Western blots for positives and negative samples were scanned together as a set of 6.
- Scans for each of these sets of Western blots are provided below representing the full gels.
- The control for each gel is the cellular prion protein blotted from the normal brain homogenate loaded in the final well of each gel.
- Gel 6 was omitted from the final version figure #1 as this was a repeat of the positive and negative controls provided on Gel #5.
- 6 blots were prepared and scanned together. The full-length scans for the positive and negative samples are provided below.

Full length Gel scans for PMCA Round #3 (positive samples plus controls).

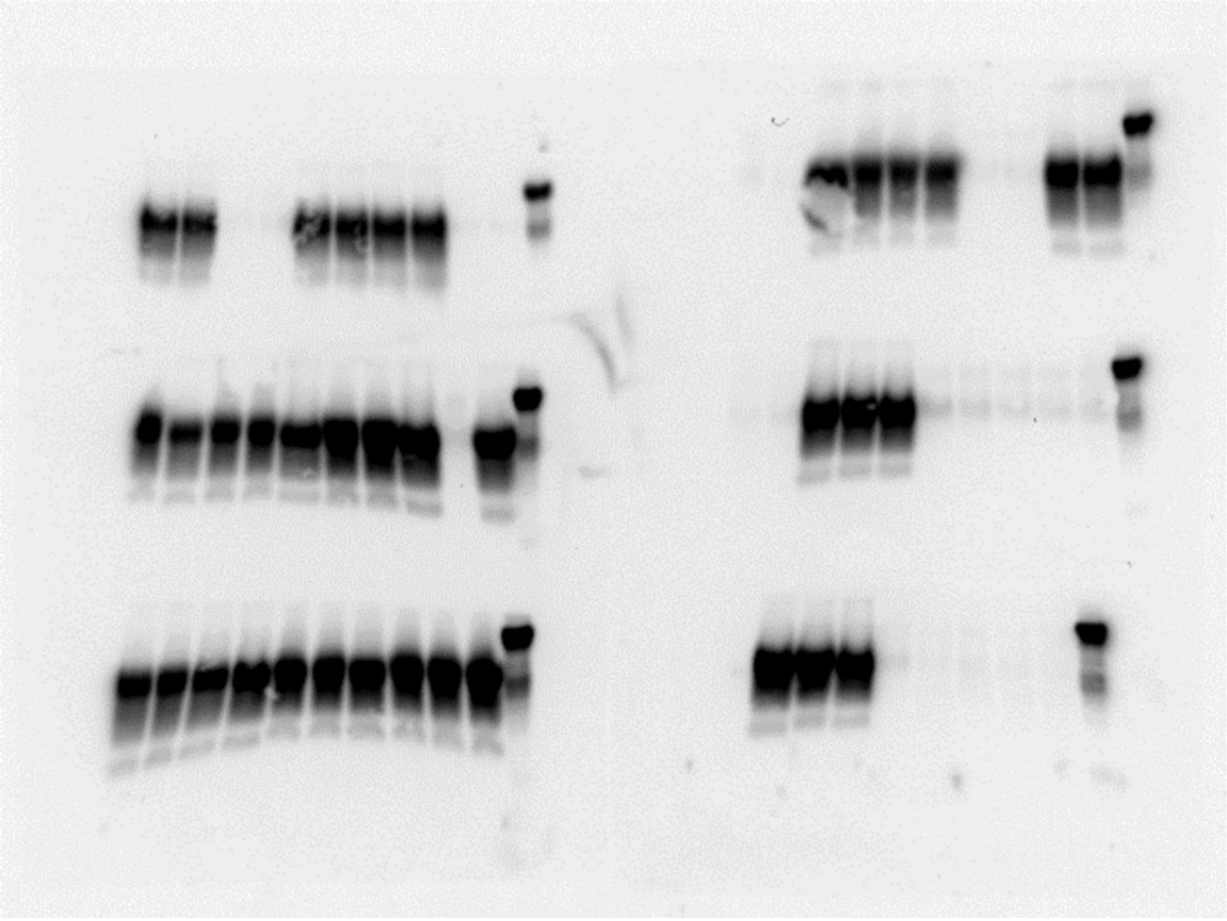

Full length Gel scans for PMCA Round #3 (negative samples).

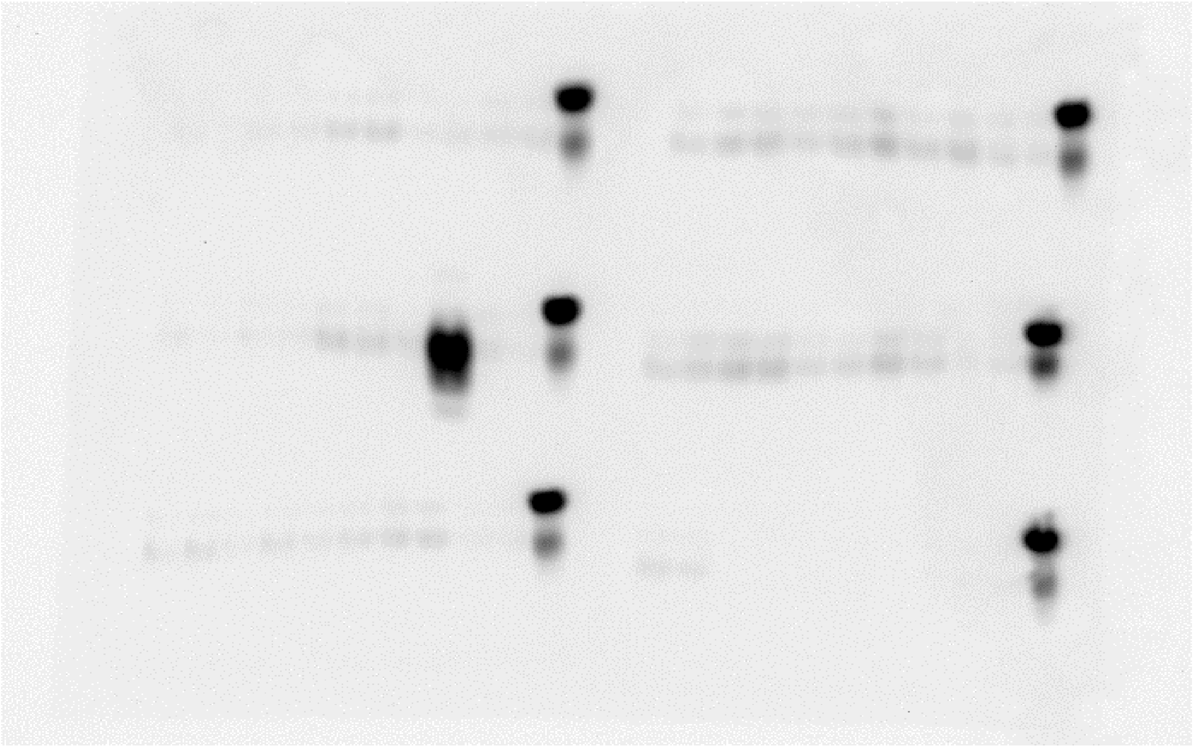

Supplementary Figure 1. The choice of human or mouse reference annotation does not influence serum miRNA abundance in elk. The mean log2 transformed normalized read count was plotted for 367 miRNAs detected in 70 elk serum samples using the human (GRCh38) and mouse (GRCm38) reference genome and accompanying miRBase annotation.

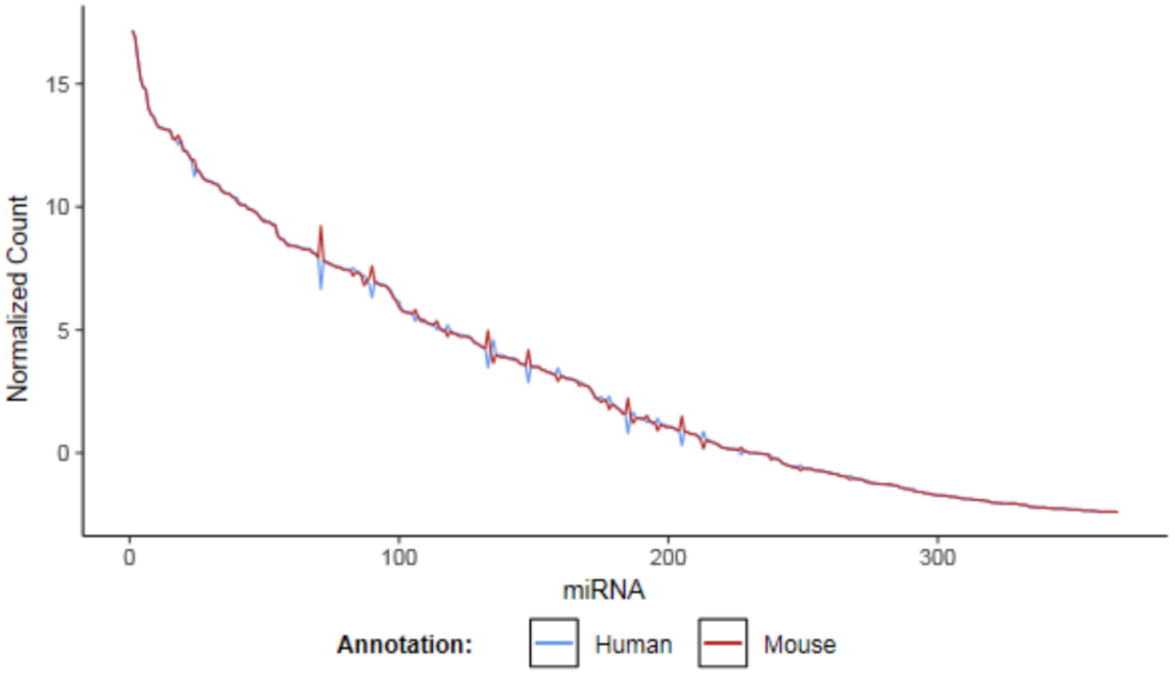

Supplementary Figure 2. Mapping statistics of elk and hamster sequencing data. The mapping statistics are shown for serum miRNA next-generation sequencing reads in the A) Elk and B) hamsters used in this study. C) The hamster serum samples had relatively high miR-451a/miR-23a-3p ratios.

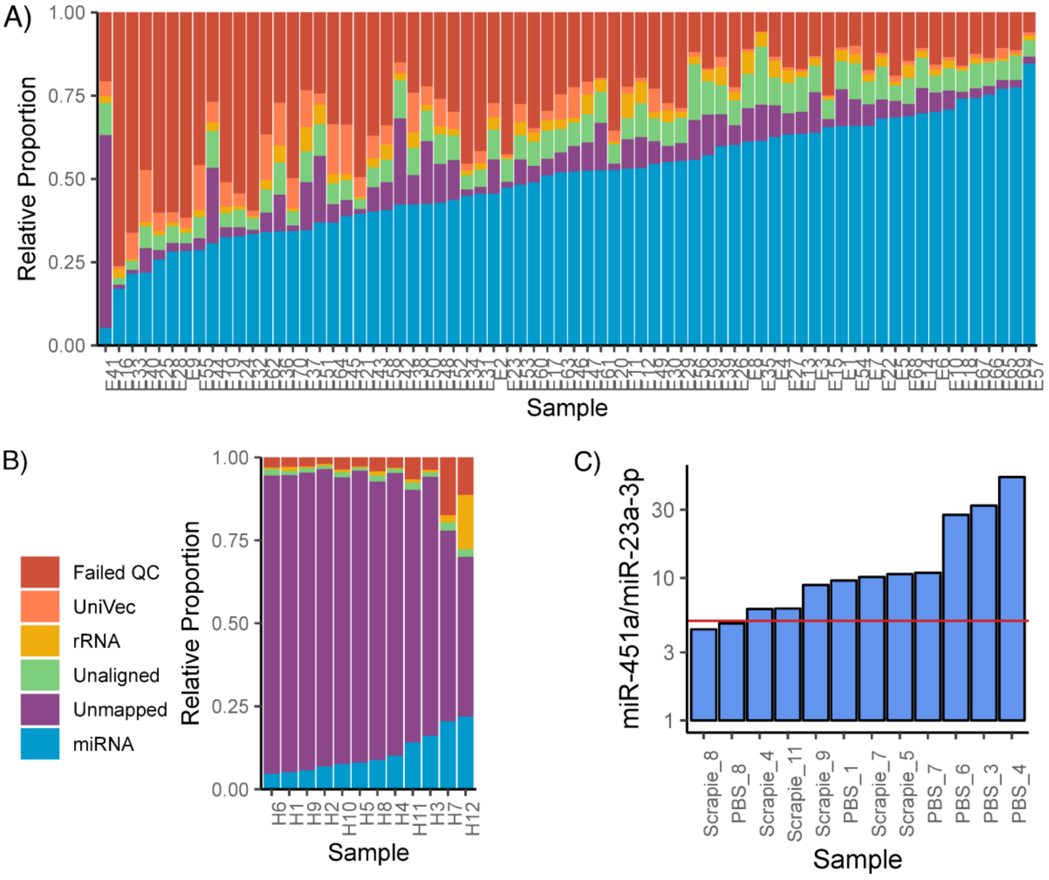

Supplementary Figure 3. Hierarchical clustering (A) and PCA (B) of the putative 21 serum miRNA biomarkers shows no relationship between IHC Scores reflecting prion staining intensity and individual elk samples.

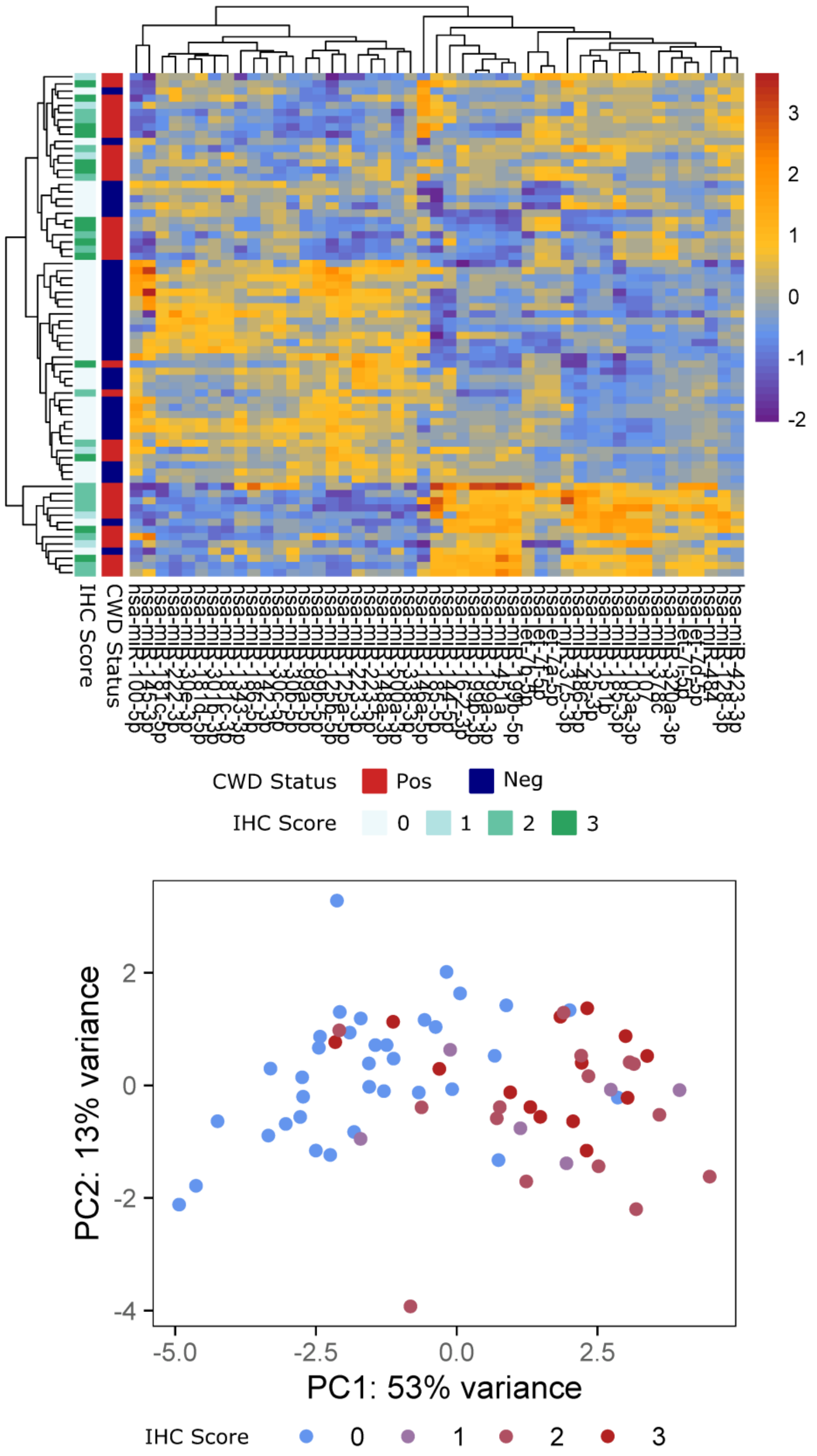

Supplementary Figure 4. KEGG pathway for prion disease on which is marked those genes that are targeted by miRNAs altered in abundance in CWD IHC positive elk. MiRNAs marked in blue are those determined using the Pathway Union tool of miRPath v3.0 that identifies the significantly targeted pathways by the selected miRNA group, whereas those in green are all genes targeted by at least one selected miRNA within the pathway. Prion Diseases pathway map was retrieved with permission from KEGG, which was developed by Kanehisa Laboratories (See refs. 49-51 in main text).

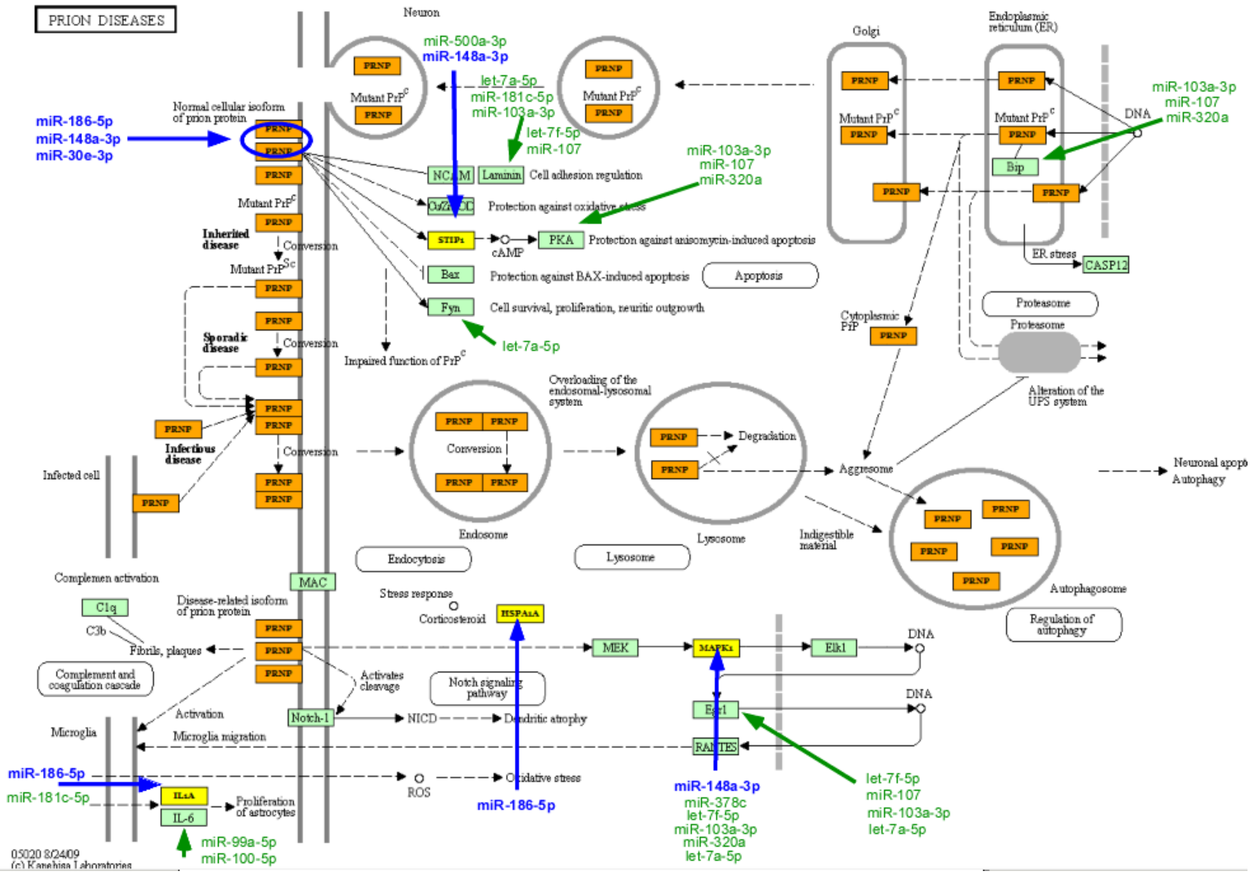

Supplement: Supplementary file 1 — Supplementary Data [file 41598_2019_56249_MOESM1_ESM.pdf]
